# Dietary Zn Deficiency Inhibits Cell Proliferation via the GPR39-Mediated Suppression of the PI3K/AKT/mTOR Signaling Pathway in the Jejunum of Broilers

**DOI:** 10.3390/ani14060979

**Published:** 2024-03-21

**Authors:** Yangyang Hu, Ke Yang, Weiyun Zhang, Mengxiao Xue, Tingting Li, Shengchen Wang, Xiaoyan Cui, Liyang Zhang, Yun Hu, Xugang Luo

**Affiliations:** 1 Poultry Mineral Nutrition Laboratory, College of Animal Science and Technology, Yangzhou University, Yangzhou 225009, China; 18200530679@163.com (Y.H.); mz120211421@stu.yzu.edu.cn (M.X.);; 2 College of Animal Science and Technology, Hebei Normal University of Science and Technology, Qinhuangdao 066004, China; mttclydk@163.com; 3 Mineral Nutrition Research Division, State Key Laboratory of Animal Nutrition, Institute of Animal Science, Chinese Academy of Agricultural Sciences, Beijing 100193, China

**Keywords:** dietary Zn deficiency, broiler, jejunum, cell proliferation, signaling pathway

## Abstract

**Simple Summary:**

Zinc is an essential mineral element for broiler growth and development. In the present study, we found that dietary Zn deficiency decreased the number of proliferating cell nuclear antigen-positive cells and inhibited PI3K, AKT, and mTOR phosphorylation along with the downregulation of GPR39 protein expression in the jejunum of broiler on d 42. These findings suggest that dietary Zn deficiency inhibits cell proliferation possibly via the GPR39-mediated suppression of the PI3K/AKT/mTOR signaling pathway in the jejunum of broilers.

**Abstract:**

A prior investigation revealed that a lack of Zinc (Zn) could hinder intestinal cell proliferation in broiler chickens; however, the mechanisms responsible for this effect remain unclear. We aimed to investigate the possible mechanisms of dietary Zn deficiency in inhibiting the jejunal cell proliferation of broilers. For this study, a total of 112 chickens (21 days old) were randomly divided into two treatments (seven replicate cages per treatment, eight chickens per replicate cage): the control group (CON) and the Zn deficiency group. The duration of feeding was 21 d. Chickens in the control group were provided with a basal diet containing an extra addition of 40 mg Zn/kg in the form of Zn sulfate, whereas chickens in the Zn deficiency group were given the basal diet with no Zn supplementation. The results indicated that, in comparison to the CON, Zn deficiency increased (*p* < 0.05) the duodenal and jejunal crypt depth (CD) of broilers on d 28 and jejunal and ileal CD on d 35, and decreased (*p* < 0.05) the duodenal, jejunal, and ileal villus height/crypt depth (VH/CD) on d 28 and the jejunal VH, jejunal and ileal villus surface area, and VH/CD on d 35. Furthermore, Zn deficiency decreased (*p* < 0.0001) the number of proliferating cell nuclear antigen-positive cells and downregulated (*p* < 0.01) the mRNA or protein expression levels of phosphatidylinositol 3-kinase (PI3K), phosphorylated PI3K, phosphorylated serine–threonine kinase (AKT), phosphorylated mechanistic target of rapamycin (mTOR), G protein-coupled receptor 39 (GPR39), and extracellular-regulated protein kinase, but upregulated (*p* < 0.05) the mRNA or protein expression levels of P38 mitogen-activated protein kinase, c-jun N-terminal kinase (JNK) 1 and JNK2, and phosphorylated protein kinase C in the jejunum of the broilers on d 42. It was concluded that dietary Zn deficiency inhibited cell proliferation possibly via the GPR39-mediated suppression of the PI3K/AKT/mTOR signaling pathway in the jejunum of broilers.

## 1. Introduction

Microelement zinc (Zn), is an essential nutrient for broiler health [1]. Prior studies have shown that a lack of Zn may result in hindered growth performance [2], altered intestinal flora composition [3], impaired immune response [4], changed bone histomorphometry [5], and reduced meat quality in chickens [6]. In addition, dietary Zn deficiency was also reported to disturb the intestinal barrier of mice [7]. Cell proliferation is crucial for preserving the integrity and function of the small intestine [8]. The number of proliferating cell nuclear antigen (PCNA)-positive cells is considered to be a biomarker of cell proliferation in various tissues [9]. Shao et al. [10] reported that Zn deficiency decreased PCNA-positive cells in the Caco-2 cells of mice. Zhang et al. [11] reported that Zn could maintain the normal barrier function of the small intestine mainly by promoting the proliferation of porcine small intestinal epithelial cells [11]. Therefore, Zn deficiency causes integrity disruption and intestinal barrier dysfunction by affecting cell proliferation. However, the molecular mechanism through which Zn deficiency inhibits cell proliferation in the small intestine is still unknown. 

It is reported that intestinal cell proliferation can be regulated by the phosphatidylinositol 3-kinase/protein kinase B/mechanistic target of rapamycin (PI3K/AKT/mTOR), protein kinase C (PKC), and mitogen-activated protein kinase (MAPK) signaling pathways [12,13,14,15]. Previous studies indicated that Zn could promote the proliferation of mouse C2C12 myoblasts, human gastric carcinoma cells, or porcine intramuscular adipocytes by activating the PI3K/AKT signaling pathway [13,14,16]. Kaltenberg et al. [17] reported that Zn-accelerated mouse T-cell proliferation depended on extracellular-regulated protein kinase (ERK) activation [17]. Liang et al. [15] found that Zn could enhance the mouse osteogenetic capabilities by stimulating the proliferation of osteoblasts via the activation of PKC and MAPK signaling pathways. Furthermore, G protein-coupled receptor 39 (GPR39), a receptor that senses Zn^2+^, plays a crucial role in controlling AKT, ERK, and PKC signaling pathways and is responsible for the majority of Zn’s biological effects. Therefore, GPR39 has the ability to detect alterations in extracellular Zn^2+^ levels and facilitate the transmission of Zn^2+^ signals [18]. In HT29 cells, GPR39 knockout attenuated Zn^2+^-dependent AKT/mTOR and ERK1/2 activation [19]. In Caco-2 cells, PKC expression was suppressed after the silencing of GPR39 [20]. The aforementioned results imply that the suppression of cell proliferation caused by a lack of Zn might be associated with the GPR39-facilitated PI3K/AKT/mTOR, MAPK, or PKC signaling pathways. However, no studies related to the above-mentioned molecular mechanisms of Zn action in mouse and in vitro cell models have been reported in broilers and other avian species before. 

Considering that the jejunum has been shown to be the most sensitive in response to Zn deficiency among the small intestinal segments of broilers [21], it was hypothesized that dietary Zn deficiency in broiler chickens would inhibit the jejunal cell proliferation of broilers possibly through the GPR39-mediated PI3K/AKT/mTOR, MAPK, and PKC signaling pathways. Hence, in order to confirm the above hypothesis, this research was designed to determine the effect of dietary Zn deficiency on the histomorphology of the small intestine, cell proliferation, and mRNA and protein abundances of GPR39 and the target genes involved in the aforementioned signaling pathways in the jejunum of broiler chickens.

## 2. Materials and Methods 

The study’s experimental protocols were carried out in compliance with the regulations set by the Animal Care Advisory Committee (Jiangsu, China) at Yangzhou University, with permit number SYXK (Su) 2021-0027.

### 2.1. Animals, Experiment Design, Treatments, and Diets 

One hundred and forty male broilers of the Arbor Acres (AA) breed (1 day old) were acquired from Jiangsu Jinghai Poultry Group Co., Ltd. (Nantong, China). During 1–21 d, the broilers were provided with a complete diet consisting of corn and soybean meal. The diet was analyzed to contain 84.40 mg of Zn per kilogram and was formulated to meet or exceed the nutritional needs of starter broilers for all nutrients recommended by the National Research Council (NRC, 1994) and the Feeding Standard of Chicken in China (2004) [22,23] (Table 1). The Zn level in the diet was decided based on our previous study by Huang et al. [24]. At 22 days of age, a total of 112 broilers were chosen based on their average body weight. These broilers were then divided into two treatment groups using a completely randomized design, with each group consisting of 7 replicate cages. Each replicate cage had 8 chickens. Broilers were kept under a 24 h constant light schedule and given unrestricted access to experimental diets and tap water containing an undetectable Zn. Broilers were fed a Zn-unsupplemented corn–soybean basal diet (Zn deficiency group) and the basal diet supplemented with 40 mg Zn/kg as Zn sulfate (reagent grade, Sinopharm Chemical Reagent Co., Ltd., Shanghai, China) (the control group, CON) for 21 d. The supplemental level of 40 mg Zn/kg as Zn sulfate was designed based on the study of Liao et al. [25]. The basal diet was formulated to meet or exceed the requirements for all nutrients except for Zn as recommended by NRC (1994) and the Feeding Standard of Chicken in China (2004) [22,23] (Table 1). The contents of Zn in the Zn deficiency group diet and the control group diet were measured to be 22.23 and 64.36 mg/kg, respectively. 

### 2.2. Sample Collections and Preparations

Samples of the diets were gathered and examined for contents of calcium, Zn, and crude protein. Tap water was collected for the analysis of Zn content. At 28, 35, and 42 days of age, all chickens were subjected to overnight fasting, but water was available ad libitum. At the end of the fasting period, one chicken with a body weight close to the cage’s average weight was selected from each replicate cage at 28 and 35 days of age, respectively, while three chickens with a body weight close to the cage’s average weight were selected from each replicate cage at 42 days of age. Then, the selected chickens were anesthetized by the intraperitoneal injection of propofol (20 mg/kg body weight), and killed by cervical dislocation. At 28 or 35 days of age, 2 cm segments in the middle of the duodenum, jejunum, and ileum of the chickens were cut and fixed in 4% paraformaldehyde to analyze the small intestine histological morphology. At 42 days of age, a 2 cm segment in the middle of the jejunum of one of the three killed chickens was cut and fixed in 4% paraformaldehyde to analyze the number of proliferating cell nuclear antigens (PCNAs), and then the mucosa of the remaining part of the jejunum and the jejunum of the remaining two chickens were scraped, packed into 1.5 mL centrifuge tubes, snap-frozen with liquid nitrogen, and placed in a chamber at −80°C for the analyses of mRNA and protein expression levels.

### 2.3. Analyses of Dietary Crude Protein, Calcium and Zn Contents, and Zn Concentration in Tap Water

We employed the Kjeldahl method to determine the crude protein contents in diets [26]. To determine calcium and Zn contents in the diets and Zn concentration in tap water, a plasma optical emission spectrometer, namely 5110 ICP-OES (Agilent Technologies Australia (M) Pty Ltd., Mulgrave, Australia) was employed. 

### 2.4. Measurements of the Small Intestine’s Histological Morphology

The small intestine’s histological morphology was measured as described previously [27,28]. Briefly, formalin-fixed intestinal samples were prepared using paraffin embedding techniques. Consecutive sections (5 mm) were stained using hematoxylin and eosin and observed for histomorphology. The villous height (VH) and crypt depth (CD) of each sample were measured from 15 randomly selected villi and associated crypts. The VH/CD ratio was then calculated from the aforementioned measurements. Villus surface areas (VSAs) were computed using the following formula: VSA = 2π (VH) × (villus width/2). For villus width, the distance between the bases of the villi (the junction of the villi to intestinal glands) was calculated. All examinations and measurements were performed with an Olympus optical microscope using Mingmei software, version 9 (Guangzhou, China).

### 2.5. Analysis of the Jejunal PCNA-Positive Cells

The jejunal specimens were subjected to H&E staining followed by their visualization under an Olympus CKX53 light microscope (Tokyo, Japan). The number of PCNA-positive cells in the jejunum was analyzed by immunohistochemistry [29]. In short, the slides underwent treatment with an antigen repair solution for a period of 10 min. After being exposed to 3% hydrogen peroxide, the slides were blocked with goat serum for a duration of 30 min. Subsequently, the slides were placed in a humid chamber at 4 °C and incubated overnight with the primary antibody, which had been diluted. On the following day, the slides were then treated with a secondary antibody and incubated for 2 h at room temperature. Afterward, the slides underwent staining with 3,3′-diaminobenzidine (DAB) and hematoxylin. Lastly, the slides were affixed with a neutral resin glue, overlaid with coverslips, and observed under an Olympus CKX53 light microscope (Tokyo, Japan) at a 100× magnification. The average optical density (AOD) value was used to reflect the number of PCNA-positive cells, and the higher the AOD value, the higher the number of PCNA-positive cells. The calculation formula of the AOD value was AOD = IOD/area, where IOD stands for the integrated optical density, which is the total responsive strength of all PCNA-positive cells, and the area is one in which all PCNA-positive cells are located within the whole selected photographed field. Five fields per chamber were photographed under a light microscope for quantification (100×).

### 2.6. RT-qPCR 

The total RNA from the jejunum was isolated using the Vazyme RNA Extraction Kit. SYBR Green RT-qPCR was used to determine the mRNA expression levels in the jejunum, with glyceraldehyde-3-phosphate dehydrogenase (GAPDH) and β-actin serving as reference genes [30]. Values of mRNA abundance levels of target genes were calculated as the relative quantities (RQ) of *PI3K*, *AKT*, *mTOR*, *P38 MAPK*, c-jun N-terminal kinase (*JNK*)*1*, *JNK2*, *ERK*, *PKC*, or *GPR39* mRNA to the geometric mean of internal reference genes *β-actin* and *GAPDH* mRNA using the 2^−^**^∆∆^**^Ct^ method [31]. All primer sequences are listed in Table 2.

### 2.7. Western Blot

The Western blot technique was carried out based on a previous study conducted by Hu et al. [32]. A Total Protein Extraction Kit (Beyotime, Shanghai, China) was used for the extraction of jejunal total proteins. To determine the protein concentration, a BCA Protein Detection Kit (ThermoFisher, Waltham, MA, USA) was employed. Each sample was diluted to a final concentration of 4 µg/µL using RIPA lysis buffer. SDS-PAGE was employed to transfer the proteins onto the NC membrane, which was then blocked with 5% skim milk for 2 h at room temperature and exposed to the primary antibody overnight at 4 °C. Subsequently, the NC membrane was rinsed with TBST, followed by a 2 h incubation with the secondary antibody. The images were captured using the AllDoc × system (Tanon). Values of expression levels of target proteins (PI3K, phosphorylated PI3K (p-PI3K), AKT, p-AKT, mTOR, p-mTOR, P38MAPK, p-P38MAPK, ERK, p-ERK, JNK, p-JNK, PKC, p-PKC, and GPR39) were calculated as the relative quantities (RQs) of the target protein band intensity to the internal reference β-actin band intensity. 

### 2.8. Statistical Analyses

Statistical analyses were performed using SAS software (version 9.4, 2013). All of the indicators (the intestinal histological morphology-related parameters (VH, CD, VH/CD, and VSA) in the duodenum, jejunum, and ileum of broilers on d 28 and d 35; PI3K, AKT, mTOR, P38MAPK, ERK, JNK1 and 2, PKC, and GPR39 mRNA levels in the jejunum of the broilers on d 42; PI3K, p-PI3K, AKT, p-AKT, mTOR, p-mTOR, P38MAPK, p-P38MAPK, ERK, p-ERK, JNK, p-JNK, PKC, p-PKC, and GPR39 protein levels in the jejunum of the broilers on d 42) measured in this study were analyzed for significant differences between the two treatment groups using student’s *t*-test, with statistical significance being defined as *p* ≤ 0.05. 

## 3. Results

### 3.1. Growth Performance

It was observed that dietary Zn deficiency had no impact (*p* > 0.05) on the average daily feed intake, the average daily gain, and the mortality of the broilers compared to the CON. However, it did lead to an increase (*p* < 0.05) in the feed/gain ratio of the broilers from d 22 to d 42. These data have been published in our previous paper [33]. 

### 3.2. Intestinal Histological Morphology

As shown in Table 3, compared with the CON, Zn deficiency increased (*p* < 0.05) the duodenal and jejunal CD but decreased (*p* < 0.05) the duodenal, jejunal and ileal villus height/crypt depth (VH/CD) of the broilers on d 28. However, Zn deficiency did not affect (*p* > 0.05) the duodenal, jejunal, and ileal VH, VSA, and ileal CD of the broilers on d 28. Compared with the CON, Zn deficiency increased (*p* < 0.05) the jejunal and ileal CD but decreased (*p* < 0.05) the jejunal VH, jejunal and ileal VH/CD, and VSA of the broilers on d 35. However, Zn deficiency did not affect (*p* > 0.05) the duodenal and ileal VH, VSA, and ileal CD of the broilers on d 35. 

### 3.3. The Number of PCNA-Positive Cells

As shown in Table 4, compared with the CON, Zn deficiency decreased (*p* < 0.0001) the number of PCNA-positive cells in the jejunum of the broilers on d 42. 

### 3.4. mRNA Expression Levels

As shown in Table 5, compared with the CON, Zn deficiency downregulated (*p* < 0.01) the mRNA expression levels of *PI3K*, *ERK*, and *GPR39* and upregulated (*p* < 0.05) the mRNA expression levels of P38 mitogen-activated protein kinase (*P38 MAPK*), c-jun N-terminal kinase 1 (*JNK1*) and *JNK2* in the jejunum of the broilers on d 42. However, Zn deficiency did not affect (*p* > 0.05) the mRNA expression levels of *AKT*, *mTOR*, and *PKC* of the broilers on d 42.

### 3.5. Protein Expression Levels

As shown in Table 6, compared with the CON, Zn deficiency decreased (*p* < 0.05) protein abundances of phosphorylated PI3K (p-PI3K), p-AKT, p-mTOR, and GPR39 in the jejunum of the broilers on d 42. However, Zn deficiency did not affect (*p* > 0.05) the protein abundances of PI3K, AKT, mTOR, P38 MAPK, p-P38 MAPK, JNK, p-JNK, ERK, p-ERK, and PKC.

## 4. Discussion

In the present study, we revealed that dietary Zn deficiency disrupted the small intestinal structure and decreased the number of PCNA-positive cells, as well as the mRNA or protein expression levels of PI3K, ERK, GPR39, p-PI3K, p-AKT, and p-mTOR, but increased the mRNA or protein expression levels of P38 MAPK and p-PKC in the jejunum of the broilers on d 42. These results indicate that dietary Zn deficiency inhibited the jejunal cell proliferation of the broilers possibly through the GPR39-mediated PI3K/AKT/mTOR signaling pathway, thereby impairing the small intestinal integrity and barrier function, which supports our hypothesis. These findings have not been reported before and provide new insights into dietary rational Zn addition in broiler production in order to improve the intestinal health of broilers.

The small intestine is a vital organ in the digestive system, where most nutrient absorption occurs. Previous studies have demonstrated that Zn deficiency had a negative impact on the villi structure of the small intestine of broilers [1,33]. The destruction of the intestinal physical barrier can cause nutrient absorption deficiency and thus affect the growth performance of animals. Intestinal morphology indexes, such as VH, VSA, CD, and the VH/CD ratio, are commonly used indices to assess intestinal development and barrier function [34]. A previous study in our laboratory indicated that Zn deficiency decreased the jejunal VH and VSA and increased the duodenal and jejunal CD, but it decreased the duodenal, jejunal, and ileal VH/CD ratios of the broilers on d 42 [33]. In the present research, we found that Zn deficiency increased the duodenal and jejunal CD and decreased the duodenal, jejunal, and ileal VH/CD ratios of the broilers on d 28, while Zn deficiency increased the ideal CD and decreased the jejunal VH, jejunal and ileal VSA, and VH/CD of the broilers on d 35. In broilers, the injury of the small intestine was reported to be related to its immune function [35]. In a previous study, it was found that Zn deficiency could lower the CD of the duodenum in broilers, accompanied by a increase in the immune response [36]. The above results indicate that dietary Zn deficiency impairs the small intestinal histomorphology, which may affect the small intestinal integrity and immune function. 

Wu et al. [33] demonstrated that the damage caused by Zn deficiency was more obvious on the jejunum of the broilers. Moreover, in our present study, dietary Zn deficiency caused more serious damage to the jejunum of the broilers. Therefore, the jejunum of the broilers on d 42 was used for follow-up examinations. Cell proliferation plays an important role in maintaining intestinal integrity and barrier function [7]. The PCNA is considered to be a marker of cell proliferation in various tissues [9]. Shao et al. [10] and Kang et al. [37] reported that Zn could increase the number of PCNA-positive cells in the Caco-2 cells or mice. Similar results were observed in the current study in which Zn deficiency reduced the number of PCNA-positive cells in the jejunum of the broilers, suggesting that Zn deficiency would impair intestinal integrity and barrier function by inhibiting cell proliferation.

Previous studies have established the vital role of the PI3K/AKT/mTOR and PKC signaling cascade in cellular growth and proliferation in mammals [38,39]. PI3K plays a crucial role as a messenger in cell proliferation and survival by activating AKT, a protein serine–threonine kinase [19]. mTOR regulates cell proliferation, autophagy, and apoptosis by participating in multiple signaling pathways in human chondrocytes [40]. Dai et al. [41] reported that dieckol inhibited cell proliferation by impeding the expression of PI3K, AKT, and mTOR. Ohashi et al. [13] found that Zn accelerated the proliferation of C2C12 myogenic cells by boosting the phosphorylation of PI3K/Akt. PKC is a key regulator of cell proliferation, survival, and cell death [42]. Liang et al. [15] demonstrated that Zn could promote osteoblast proliferation by activating the PKC signaling pathway. Similar results were found in the current study in which dietary Zn deficiency decreased the cell proliferation associated with the downregulation of p-PI3K, p-AKT, and p-mTOR protein expressions. However, the protein expression of p-PKC in the jejunum of the broilers was increased by Zn deficiency, which is different from a previous study in 3T3 cells [43], which showed a downregulated p-PKC protein expression. The discrepancy might be attributed to different study models, the method used, or the dose of supplemental Zn.

GPR39 is a Zn-sensing receptor whose activation leads to the activation of the PI3K/AKT/mTOR signaling cascade in mammals [44,45]. In normal immortalized human hepatocyte LO2 and HCC cell lines, silencing GPR39 reduced the protein expressions of p-PI3K, p-AKT, and p-mTOR [45]. In the present study, we found that Zn deficiency inhibited PI3K, AKT, and mTOR phosphorylation along with the downregulation of GPR39 protein expression. The above findings suggest that Zn deficiency might inhibit the PI3K/AKT/mTOR signaling pathway by restraining GPR39 expression.

## 5. Conclusions

Dietary Zn deficiency inhibits cell proliferation possibly via the GPR39-mediated suppression of the PI3K/AKT/mTOR signaling pathway in the jejunum of broilers. Further studies need to be carried out using the primary cultured jejunal epithelial cells as well as gene overexpression and RNA interference (RNAi) to address and confirm the above possible mechanisms.

## Figures and Tables

**Table 1:** Composition and nutrient contents of the broiler diets (as-fed basis).

Ingredient	1 to 21 Days of Age	22 to 42 Days of Age	
		Control Group	Zn Deficiency Group
Corn (%)	54.75	58.78	58.78
Soybean meal (%)	36.31	32.56	32.56
Soybean oil (%)	4.95	5.15	5.15
CaHPO_4_ (%) ^a^	1.87	1.67	1.67
CaCO_3_ (%) ^a^	1.20	1.09	1.09
NaCl (%) ^a^	0.30	0.30	0.30
DL-methionine (%) ^b^	0.31	0.15	0.15
Micronutrient (%) ^c^	0.31	0.20	0.20
Corn starch (%) ^d^	0.00	0.08	0.10
ZnSO_4_·7H_2_O (%) ^a^	0.00	0.02	0.00
Total (%)	100.00	100.00	100.00
Nutrient composition			
Metabolizable energy (MJ/kg) ^e^	12.75	12.98	12.98
Crude protein (%) ^f^	21.44	20.08	20.08
Lysine (%) ^e^	1.10	1.01	1.01
Methionine (%) ^e^	0.61	0.44	0.44
L-threonine (%) ^e^	0.80	0.75	0.75
Tryptophan (%) ^e^	0.24	0.22	0.22
Methionine + cysteine (%) ^e^	0.91	0.72	0.72
Ca (%) ^f^	1.01	0.92	0.92
Non-phytate P (%) ^e^	0.45	0.40	0.40
Zn (mg/kg) ^f^	84.40	64.36	22.23

^a^ Reagent grade. ^b^ Feed grade. ^c^ Provided per kilogram of diet for d 1 to 21: vitamin A 12,000 IU; vitamin D_3_ 4500 IU; vitamin E 33 IU; vitamin K_3_ 3 mg; vitamin B_1_ (thiamin) 3 mg; vitamin B_2_ (riboflavin) 9.6 mg; vitamin B_6_ 4.5 mg; vitamin B_12_ 0.03 mg; pantothenic acid calcium 15 mg; niacin 54 mg; folic acid 1.5 mg; biotin 0.15 mg; choline 700 mg; Cu (CuSO_4_·5H_2_O) 6 mg; Fe (FeSO_4_·7H_2_O) 40 mg; Zn (ZnSO_4_·7H_2_O) 60 mg; Mn (MnSO_4_·H_2_O) 110 mg; Se (Na_2_SeO_3_) 0.35 mg; I (Ca(IO_3_)_2_·H_2_O) 0.35 mg; for d 22 to d 42: vitamin A 8000 IU; vitamin D_3_ 3000 IU; vitamin E 22 IU; vitamin K_3_ 2 mg; vitamin B_1_ (thiamin) 2 mg; vitamin B_2_ (riboflavin) 6.4 mg; vitamin B_6_ 3 mg; vitamin B_12_ 0.02 mg; pantothenic acid calcium 10 mg; niacin 36 mg; folic acid 1.0 mg; biotin 0.10 mg; choline 500 mg; Cu (CuSO_4_·5H_2_O) 6 mg; Fe (FeSO_4_·7H_2_O) 30 mg; Mn (MnSO_4_·H_2_O) 80 mg; Se (Na_2_SeO_3_) 0.35 mg; I (Ca(IO_3_)_2_·H_2_O) 0.35 mg. ^d^ ZnSO_4_·7H_2_O added in place of the equivalent weight of corn starch to produce the control group diet. ^e^ Calculated values. ^f^ Values determined by analysis, and each value is based on triplicate determinations.

**Table 2:** Primer sequences for real-time PCR amplification *.

Genes	GenBank ID	Primer Sequences	Product Length (bp)
*PI3K*	XM_046923916.1	F: 5′-CTTCTGGAGTCCTATTGTCG-3′	132
		R: 5′-CACCTTCTGGGTCTCATCTT-3′	
*AKT*	NM_205055	F: 5′-GCCGTGAGCCCAGTTAGG-3′	153
		R: 5′-AGCTACTTATGGCTGCGGGA-3′	
*mTOR*	XM_417614.6	F: 5′-AACCACTGCTCGCCACAATGC-3′	120
		R: 5′-CATAGGATCGCCACACGGATTAGC-3′	
*P38 MAPK*	XM_040691290.1	F: 5′-ACGTGCAGTTCCTCATATACCA-3′	145
		R: 5′-TGTCGAGCCAAGCCAAAATC-3′	
*ERK*	NM_204150.1	F: 5′-TCTTACTGCGCTTCAGGCAT-3′	158
		R: 5′-AATGTGGTCGTTGCTGAGGT-3′	
*JNK1*	XM_040675398.1	F:5′-GCTGGTTATAGACGCCTCGA-3′	137
		R: 5′-GCTCCCTCTCATCTAACTGCT-3′	
*JNK2*	NM_001396829.1	F: 5′-AGAATCAAACCCACGCAAAA-3′	148
		R: 5′-ATCAGTTCCATAACCAAATA-3′	
*PKC*	NM_001012804.2	F: 5′-GGCGGACAGGAAGAATACAGAGG-3′	146
		R: 5′-GAAGCTGTGTCAGGAATGGTGGTT-3′	
*GPR39*	NM_001080105.1	F: 5′-GCTGTAAAGATTGGTAAGCACTGA-3′	151
		R: 5′-ATATGCACAAGTCTGAGCGGT-3′	
*β-actin*	NM_205518.1	F: 5′-CAGCCATCTTTCTTGGGTAT-3′	169
		R: 5′-CTGTGATCTCCTTCTGCATCC-3′	
*GAPDH*	NM_204305.1	F: 5′-CTTTGGCATTGTGGAGGGTC-3′	128
		R: 5′-ACGCTGGGATGATGTTCTGG-3′	

* *PI3K*, phosphatidylinositol 3-kinase; *AKT*, serine–threonine kinase; *mTOR*, target of rapamycin; *P38MAPK*, P38 mitogen-activated protein kinase; *ERK*, extracellular-regulated protein kinase; *JNK*, c-jun N-terminal kinase; *PKC*, protein kinase C; *GPR39*, G protein-coupled receptor 39. *GAPDH*, glyceraldehyde-3-phosphate dehydrogenase; F, forward; R, reverse.

**Table 3:** Effect of dietary Zn deficiency on histological morphology of the small intestine in broilers at 28 and 35 days of age.

Intestinal Region		D 28				D 35			
		CON ^1^	Zn Deficiency Group ^1^	SEM	*p*-Value	CON ^1^	Zn Deficiency Group ^1^	SEM	*p*-Value
Duodenum	Villus height (μm)	2071	1939	54.8	0.177	1612	1546	26.0	0.133
	Crypt depth (μm)	199 ^b^	218 ^a^	3.10	0.003	169	166	2.54	0.534
	Villus height/crypt depth	10.35 ^a^	8.96 ^b^	0.261	0.008	9.70	9.30	0.170	0.282
	Villus surface area (mm^2^)	1.299	1.264	0.031	0.555	1.135	1.113	0.032	0.734
Jejunum	Villus height (μm)	1736	1577	96.4	0.282	1521 ^a^	1303 ^b^	43.0	0.029
	Crypt depth (μm)	186 ^b^	208 ^a^	11.89	0.031	168 ^b^	180 ^a^	0.703	0.050
	Villus height/crypt depth	9.65 ^a^	7.73 ^b^	0.207	0.032	8.73 ^a^	7.39 ^b^	0.276	0.009
	Villus surface area (mm^2^)	1.030	0.818	0.072	0.066	0.936 ^a^	0.731 ^b^	0.026	0.009
Ileum	Villus height (μm)	910	871	18.9	0.324	819	770	13.7	0.241
	Crypt depth (μm)	185	213	10.19	0.084	158 ^b^	175 ^a^	0.644	0.001
	Villus height/crypt depth	5.16 ^a^	4.29 ^b^	0.222	0.020	5.21 ^a^	4.43 ^b^	0.080	0.002
	Villus surface area (mm^2^)	0.405	0.360	0.016	0.254	0.504 ^a^	0.400 ^b^	0.008	0.003

^1^ Values are the means of 5–7 replicate cages of 1 bird per replicate cage (*n* = 5–7). ^a,b^ Means with different superscripts within the same row differ (*p* ≤ 0.05).

**Table 4:** Effect of dietary Zn deficiency on the number of PCNA-positive cells in the jejunum of broilers at 42 days of age.

Groups	PCNA-Positive Cells (AOD Value)
CON ^1^	0.3591 ^a^
Zn deficiency group ^1^	0.1235 ^b^
SEM	0.0034
*p*-value	<0.0001

^1^ Values are the means of 7 replicate cages of 1 bird per replicate cage (*n* = 7). ^a,b^ Means with different superscripts within the same column differ (*p* < 0.05).

**Table 5:** Effect of dietary Zn deficiency on mRNA expression levels of target genes involved in the cell proliferation-related signaling pathways in the jejunum of broilers at 42 days of age.

Groups	CON ^1^	Zn Deficiency Group ^1^	SEM	*p*-Value
*PI3K* ^2^ mRNA	1.209 ^a^	0.970 ^b^	0.0658	<0.0001
*AKT* ^2^ mRNA	1.011	1.016	0.0581	0.9598
*mTOR* ^2^ mRNA	0.997	1.009	0.0492	0.8739
*P38 MAPK* ^2^ mRNA	0.621 ^b^	1.003 ^a^	0.0375	<0.0001
*JNK1* ^2^ mRNA	0.826 ^b^	1.006 ^a^	0.0454	0.0254
*JNK2* ^2^ mRNA	0.728 ^b^	1.004 ^a^	0.0300	0.0002
*ERK* ^2^ mRNA	1.210 ^a^	1.004 ^b^	0.0358	0.0018
*PKC* ^2^ mRNA	1.020	1.002	0.0264	0.7378
*GPR39* ^2^ mRNA	1.669 ^a^	1.004 ^b^	0.0360	<0.0001

^1^ Values are the means of 5–7 replicate cages of 3 broilers per replicate cage) (*n* = 5–7). ^2^ *PI3K*, phosphatidylinositol 3-kinases; *AKT*, protein–serine–threonine kinase; *JNK*, c-jun N-terminal kinase; *P38 MAPK*, mitogen-activated protein kinase; *mTOR*, mechanistic target of rapamycin; *ERK*, extracellular-regulated protein kinase; *PKC*, protein kinase C; *GPR39*, G protein-coupled receptor 39. ^a,b^ Means with different superscripts within the same row differ (*p* < 0.05).

**Table 6:** Effect of dietary Zn deficiency on protein expression levels of target proteins or phosphorylated proteins involved in the cell proliferation-related signaling pathways in the jejunum of broilers at 42 days of age.

Groups	CON ^1^	Zn Deficiency Group ^1^	SEM	*p*-Value
PI3K ^2^	0.59	0.54	0.0111	0.0717
p-PI3K ^2^	0.48 ^a^	0.29 ^b^	0.0102	0.0012
AKT ^2^	0.81	0.86	0.0097	0.1414
p-AKT ^2^	0.62 ^a^	0.43 ^b^	0.0109	0.0008
mTOR ^2^	0.58	0.54	0.0203	0.2690
p-mTOR ^2^	0.33 ^a^	0.29 ^b^	0.0084	0.0213
P38 MAPK ^2^	0.64	0.65	0.0058	0.7956
p-P38 MAPK ^2^	0.48	0.48	0.0129	0.8395
JNK ^2^	0.67	0.63	0.0152	0.1793
p-JNK ^2^	0.24	0.22	0.0204	0.6197
ERK ^2^	0.57	0.58	0.0115	0.9842
p-ERK ^2^	0.51	0.46	0.0180	0.2416
PKC ^2^	0.52	0.52	0.0068	0.7377
p-PKC ^2^	0.30 ^b^	0.33 ^a^	0.0063	0.0142
GPR39 ^2^	0.84 ^a^	0.74 ^b^	0.0211	0.0081

^1^ Values are the means of 5–7 replicate cages of 3 broilers per replicate cage (*n* = 5–7). ^2^ PI3K, phosphatidylinositol 3-kinase; p-PI3K, phosphorylated phosphatidylinositol 3-kinase; AKT, serine–threonine kinase; p-AKT, phosphorylated serine–threonine kinase; mTOR, mechanistic target of rapamycin; p-mTOR, phosphorylated mechanistic target of rapamycin; P38 MAPK, P38 mitogen-activated protein kinase; p-P38 MAPK, phosphorylated P38 mitogen-activated protein kinase; ERK, extracellular-regulated protein kinase; p-ERK, phosphorylated extracellular-regulated protein kinase; JNK, c-jun N-terminal kinase; p-JNK, phosphorylated c-jun N-terminal kinase; PKC, protein kinase C; p-PKC, phosphorylated protein kinase C; GPR39, G protein-coupled receptor 39. ^a,b^ Means with different superscripts within the same row differ (*p* < 0.05).

## Data Availability

The data presented in this study are available in article.
